# Transcription Factor BnaC04.MYB89 Negatively Regulates Seed Fatty Acid Biosynthesis in *Brassica napus*

**DOI:** 10.3390/plants14223495

**Published:** 2025-11-16

**Authors:** Dong Li, Xumin Wang, Yujiao Song, Jianchao Sun, Shuhan Yu, Bowei Zhu, Xin Liu, Guodong Zhao, Tongsheng Zhao, Limin Wang, Yuting Sheng, Hongxia Zhang

**Affiliations:** 1 Yantai Technology Center of Characteristic Plant Gene Editing and Germplasm Innovation, Engineering Research Institute of Agriculture and Forestry, Ludong University, Yantai 264025, China; ld@nwafu.edu.cn (D.L.); 15939492734@163.com (Y.S.); 13515357072@163.com (S.Y.); 2 Yantai Key Laboratory of Crop Molecular Breeding for High-Yield and Stress-Resistant Crops and Efficient Cultivation, College of Horticulture, Ludong University, Yantai 264025, China; 3 Changli Institute of Pomology, Hebei Academy of Agriculture and Forestry Sciences, Qinhuangdao 066600, China; 15383822973@163.com (X.W.); 18331561549@163.com (B.Z.); 18830180711@163.com (X.L.); guodong19823@163.com (G.Z.); tshzh71@163.com (T.Z.); 4 Bureau of Agriculture and Rural Affairs of Weichang Manchu and Mongolian Autonomous County, Chengde 068450, China; 18931353275@163.com; 5 College of Agriculture and Forestry Science, Linyi University, Linyi 276000, China

**Keywords:** transcription factors, MYB89, *Brassica napus*, seed fatty acid

## Abstract

Seed oil content and fatty acid (FA) composition collectively determine the quality and economic value of *Brassica napus*. Little is known about the role of R2R3-MYB transcription factors (TFs) in regulating FA biosynthesis in *B. napus*. Here, *BnaC04.MYB89* was found to be expressed primarily in developing seeds. Overexpression of *BnaC04.MYB89* consistently decreased FA levels, as evidenced by its effect in both the *Arabidopsis thaliana myb89-1* mutant and *B. napus* seeds. RNA-seq of developing seeds at 30 DAP (days after pollination) revealed marked suppression of FA biosynthetic genes in *BnaC04.MYB89*-overexpressing plants compared to the K407 control. ChIP (Chromatin immunoprecipitation) analysis revealed that BnaC04.MYB89 directly inhibited the expression of *BnaA03.BCCP1* and *BnaC03.HD* while indirectly regulating that of *BnaA09.BADC1*, *BnaA03.BADC3*, *BnaA03.MOD1*, and *BnaA08.FAT8*, thereby reducing seed FA accumulation. Collectively, these results elucidate the role for BnaC04.MYB89 and provide new insights into the transcriptional regulatory network controlling seed oil accumulation in *B. napus*.

## 1. Introduction

*Brassica napus* is a major oil crop whose economic importance stems from both its industrial applications and, foremost, its role as a source of nutritious vegetable oil [1,2]. In modern “double low” (low in erucic acid and glucosinolates) cultivars, seeds contain 38–50% oil that is composed of a mixture of fatty acids including oleic acid (18:1, 60–65%), polyunsaturated fatty acids (18:2 and 18:3, 20–35%), and saturated fatty acids (16:0 and 18:0, 5–8%) [3,4]. As essential dietary components, linoleic and linolenic acids are vital for preventing deficiency-related disorders since they cannot be synthesized by the human body [5]. Given the nutritional value of this profile, increasing seed oil quality and oil quantity is one of the most important breeding criteria. This imperative is further amplified by the rapidly growing global demand for vegetable oils in recent years, incentivizing an enormous amount of effort to enhance rapeseed oil production [6,7]. Therefore, understanding the molecular mechanisms of seed FA biosynthesis in *B. napus* carries great ecological and social significance.

The fatty acid (FA) biosynthetic pathway has been extensively investigated and reviewed [8]. In this pathway, four major steps, namely condensation, reduction, dehydration, and another reduction, work in a coordinated manner to determine the FA composition and seed oil content in plants [9]. These reactions involve the participation of key enzymes such as acetyl-CoA carboxylase (ACCase), malonyl-CoA-ACP malonyltransferase (MCAMT), β-ketoacyl-ACP synthase (KAS), and others [9,10]. In *Arabidopsis thaliana*, the model system for investigating seed FA biosynthesis [11], a precise regulatory network of essential transcription factors (TFs) orchestrates the seed FA content by controlling the expression of genes involved in FA biosynthetic [8,12]. *WRINKLED1* (*WRI1*) functions as the central controller of FA biosynthesis by regulating enzymes participating in both late glycolytic reactions and the plastid FA biosynthetic process [13,14]. Overexpression of *LEAFY COTYLEDON1* (*LEC1*) globally induces the expression of FA biosynthetic genes, thereby substantially enhancing the production of major FA compositions and lipids [15]. *LEC2* directly modulates *WRI1* expression, thereby regulating the biosynthesis of biotin and lipoic acid [16]. *FUS3* mediates oil deposition by upregulating the expression of FA biosynthesis- and photosynthesis-related genes [17,18,19]. Removal of DELLA (Gibberellin signaling repressors) leads to upregulation of five GDSL-type *SFAR* genes that negatively regulate seed FA storage [20]. TRANSPARENT TESTA8 (TT8) negatively regulates seed FA accumulation through transcriptional repression of *LEC1*, *LEC2*, and *FUS3* [21]. Removal of TT2 upregulates the expression of *FUS3*, *BCCP2*, *KASII*, etc., thus promoting FA biosynthesis [22]. *TTG1* functions as a key downstream effector of FUS3, mediating the coordinated accumulation of seed FAs and storage proteins within the hierarchical regulatory network [23]. In addition, these genes have also been used in *B. napus* seeds to manipulate the FA biosynthetic process through genetic engineering [6,24,25,26,27,28,29].

In *A. thaliana*, MYB TFs play crucial roles in FA biosynthetic pathways. For example, AtMYB5 represses FA biosynthesis by directly regulating the expression of *AtTT8* and *AtAAD3* in seeds [30]. AtMYB73 represses seed oil accumulation by downregulating genes in the FA biosynthetic process [31]. AtMYB76 regulates numerous genes helpful for FA biosynthesis during seed development [32]. AtMYB89 represses seed FA biosynthesis by modulating a transcriptional cascade involving *AtWRI1*, *AtL1L*, and other key FA biosynthetic genes [14]. AtMYB96 enhances seed triacylglycerol biosynthesis by directly activating the expression of *AtPDAT1* and *AtDGAT1* and stimulates very long-chain FA biosynthesis by upregulating the expression of *AtFAE1* [33,34]. And loss of *AtMYB118* function leads to endosperm-specific derepression of seed maturation genes, consequently promoting storage metabolism [35]. Recently, significant progress has been made in understanding the role of MYB TFs in regulating FA accumulation in other plant species. For example, JcMYB1 in *Jatropha curcas* can alter the FA composition and increase the seed oil content in transgenic Arabidopsis and tobacco [36]. In *Chlamydomonas reinhardtii*, CrMYB1 promotes oil accumulation by mediating FA trafficking between the chloroplast and the endoplasmic reticulum [37,38]. And VfMYB36, an R2R3-MYB TF from *Vernicia fordii*, significantly elevates levels of seed oil and linolenic acid in transgenic Arabidopsis [39]. Another R2R3-MYB TF from *Elaeis guineensis* Jacq., EgMYB108, positively regulates long-chain FA accumulation in the oil palm mesocarp by directly targeting the promoters of *EgLACS4* and *EgKCS5* [40]. However, the role of these MYBs in regulating seed FA biosynthesis in *B. napus* remains largely unexplored.

Here, we found that *BnaC04.MYB89*, an R2R3-MYB gene, is strongly upregulated in developing seeds during the crucial stage of seed FA accumulation in *B. napus*. We identified BnaC04.MYB89 as a key regulator that directly binds to and indirectly modulates multiple FA biosynthesis genes during *B. napus* seed development. Our results indicate that BnaC04.MYB89 negatively regulates seed FA accumulation during seed development.

## 2. Materials and Methods

### 2.1. Plant Materials and Growth Conditions

Arabidopsis (Columbia-0 ecotype, Col-0) and *Brassica napus* (winter-type inbred line “K407”) served as the wild-type controls. The *myb89-1* knockout mutant (SALK_109375) was generated in a Col-0 background. All Arabidopsis seeds were surface-sterilized before sowing on the 1/2 Murashige and Skoog (MS) medium solidified with 1% (*w*/*v*) agar (pH 5.8–6.0). After synchronization at 4 °C for 2 days, seeds were transferred to a climate chamber at 22 °C with 16 h light/8 h dark cycles (average intensity 160 μmol/m^2^/s). The 7-day-old Arabidopsis seedling grown on the 1/2 MS medium were transplanted to a soil–vermiculite (4:1, *v*/*v*) composite substrate, and then transferred to the climate chamber, as reported previously [14]. The *B. napus* plants were cultivated under long-day (LD) conditions in the greenhouse, with 16 h natural daylight supplemented with LED lamps (average intensity 300–400 μmol/m^2^/s) at 25 °C and 8 h dark at 18 °C. After one month, the plants were vernalized at 4 °C for one month under LD conditions. Then uniform plants were selected and cultivated under LD conditions, as previously described [41].

### 2.2. Protein Sequence Analyses

MYB89 amino acid sequences were retrieved from the NCBI database (https://www.ncbi.nlm.nih.gov/ accessed on 23 February 2023). Multiple sequence alignment of MYB89 protein sequences were performed using the MUSCLE tool (http://www.ebi.ac.uk/Tools/msa/muscle/ accessed on 7 December 2024). Conserved MYB-like DNA-binding domains and DNA-contact residues of BnaA04.MYB89 were identified using the Conserved Domain Database (CDD) (https://www.ncbi.nlm.nih.gov/Structure/cdd/wrpsb.cgi accessed on 7 December 2024). The 3D structures of three MYB89 proteins were modeled using SWISS-MODEL (https://swissmodel.expasy.org/ accessed on 7 December 2024) and visualized using the Pymol software (v3.1.3).

### 2.3. Gene Cloning and Vector Construction

*BnaMYB89* genes were obtained from the BnIR database (https://yanglab.hzau.edu.cn/BnIR accessed on 23 February 2023). Due to high sequence homology observed between the two *BnaMYB89* paralogs, the Coding Sequence (CDS) of the BnaC04.MYB89 gene was amplified by nested PCR. Two distinct primers, the gene-specific and cloned primers, were synthesized for targeted amplification. The gene-specific primers were custom-designed according to the differential sites identified through sequence alignment of the two *BnaMYB89* genes, and the cloned primers were custom-designed according to the CDS of *BnaC04.MYB89* (BnaC04T0392300ZS), excluding the stop codon. RNA was isolated from developing seeds of the *B. napus* cultivar “Zhongshuang11” using the Plant Total RNA Isolation Kit (Vazyme #RC411). Subsequently, the purified RNA was reverse transcribed into cDNA using the cDNA Synthesis Kit (Vazyme #R212), and the synthesized cDNA served as the PCR template for gene cloning.

To obtain the CDS of *BnaC04.MYB89*, a two-step nested PCR strategy was employed. The primary PCR amplification step was conducted using specific primers (BnaC04.MYB89_Fs/Rs), while the secondary amplification with cloned primers enabled the successful cloning of the *BnaC04.MYB89* gene’s CDS. To generate the transformation vector for Arabidopsis, the CDS encoding *BnaC04.MYB89* without a stop codon was PCR-amplified and ligated into a pGreen-6HA vector at the *EcoR*I and *Pst*I restriction sites [42]. Similarly, the CDS encoding *BnaC04.MYB89* without a stop codon was PCR-amplified and inserted into a pCAMBIA1300-6HA vector for *B. napus* transformation. To construct the Green Fluorescent Protein (GFP) vector, the CDS encoding *BnaC04.MYB89* without a stop codon was ligated into the *EcoR*I and *Pst*I sites of a pGreen-35S-GFP vector. To generate the Pro*BnaC04.MYB89*::GUS vector, a 2.0 kb region upstream of the *BnaC04.MYB89* start codon was cloned into *Cla*I and *Spe*I sites of a pHY107 vector, which carries the β-glucuronidase (GUS) reporter gene [43].

All DNA fragments were amplified using the KOD polymerase (TOYOBO #KOD-201), cloned into the respective vectors, and sequenced with vector-specific primers (pGreen_F, pCAMBIA_F, or HY107_F) at Sangon Biotechnology (Shanghai, China). The primer sequences used for cloning DNA fragments are shown in Table S1.

### 2.4. Subcellular Localization

The *Agrobacterium tumefaciens* GV3101 strain harboring the 35S::*BnaC04.MYB89*-GFP vector was used for transient expression of the fusion protein in the 3-week-old leaves of transgenic *Nicotiana benthamiana* with a nuclear localization signal (NLS), as reported in a prior study [44]. The plants were cultured in a climate chamber under LD conditions at 22 °C. Confocal laser scanning microscopy (ZEISS LSM 900, Oberkochen, Germany) was employed to visualize the fluorescent signals 72 h after agroinfiltration.

### 2.5. Plant Transformation

To obtain transgenic *A. thaliana*, the *Agrobacterium tumefaciens* GV3101 strain harboring the construct 35S::*BnaC04.MYB89*-6HA (based on the pGreen-6HA vector) was used to transform *myb89-1* mutants, while the strain harboring the Pro*BnaC04.MYB89*::GUS vector was used to transform Col-0 plants, both via the floral-dip method [45]. T_1_-generation seeds were screened with 0.002% (*v*/*v*) Basta (Bayer #YQ1363). Then homozygous T_3_ overexpression transgenic plants (*OE1*, *OE2*, *OE3*, *OE4*, *OE5*, and *OE6*) were screened in the 1/2 MS medium containing glufosinate-ammonium (Aladdin #G114499, 10 mg/L), confirmed with PCR, and subjected to transgene expression analyses.

To generate transgenic *B. napus*, the hypocotyls of K407 were infected with the GV3101 strain harboring the construct 35S::*BnaC04.MYB89*-6HA (based on the pCAMBIA1300-6HA vector). Transformed *B. napus* overexpression lines were screened with 10 mg/L (*w*/*v*) hygromycin (Beyotime #ST1389), and the homozygous transgenic plants (Lines 3, 11, and 28) were used for further experiments.

### 2.6. RNA-Seq Experiment and Data Analysis

For RNA sequencing (RNA-seq) experiments, flowers of wild-type *B. napus* K407 and the homozygous transgenic line 11 (*OE#11*) plants marked with different colored threads represent the day after pollination (DAP). The 30 DAP seeds of K407 and homozygous transgenic line 11 (*OE#11*) were taken from the middle and base of primary inflorescence, and ten plants from each line were used for RNA-seq. The experiments were conducted at Tsingke Biological Technology (Beijing, China) following the typical protocol (https://www.tsingke.com.cn/tec/eukaryotic accessed on 12 June 2024) with 3 independent biological replicates. In short, pair-end sequencing was obtained using the NovaSeq 6000 platform, and low-quality base sequences were filtrated using the FASTP software (v0.20.0). The reference genome sequence of “Zhongshuang11” was obtained from the BnPIR database (Pan-Genome Information Resource, http://cbi.hzau.edu.cn/rape/download_ext/zs11.genome.fa accessed on 12 June 2024). RNA-seq reads were mapped to the *B. napus* genome using HISAT2 (v2.2.1). Differentially expressed genes (DEGs) were determined based on the following parameters: |log_2_ fold change| ≥ log_2_(2) and False Discovery Rate (*FDR*) ≤ 0.01. The GO annotation dataset for *B. napus* was obtained from the BnIR database (Brassica napus Information Resource, https://yanglab.hzau.edu.cn/BnIR/GO accessed on 23 November 2024), and GO analysis was performed on the DAVID database (https://davidbioinformatics.nih.gov/ accessed on 24 November 2024). DEGs with a log_2_ fold change of less than −1 or greater than 1 are shown in Tables S2 and S3.

### 2.7. Gene Expression and GUS Staining

The *B. napus* cultivar K407 was grown in the greenhouse at Ludong University, China, under 16 h natural daylight supplemented with LED lamps (average intensity 300–400 μmol/m^2^/s) at 25 °C and 8 h dark conditions at 18 °C. The RNA samples used to verify RNA-seq findings were similar to those utilized for the RNA-seq experiment. Roots, stems, leaves, flowers, and seeds of K407 were collected to analyze the stage- and tissue-specific expression patterns of *BnaC04.MYB89*. Seeds at different development stages were marked with different colored threads. DAP represent the day after pollination. Total RNA was extracted using Vazyme’s Plant Total RNA Isolation Kit (#RC411), and cDNA was synthesized using the HiScript II Q Select RT SuperMix (#R211). RT-qPCR was performed using the StepOnePlus™ RealTime PCR System (Applied Biosystems, Waltham, MA, USA) with the SYBR qPCR Master Mix (Vazyme #Q711). The expression levels of the target genes were normalized to those of *AtEF1αA4* or *BnaGAPDH* and analyzed via 2^−ΔΔCt^ [46]. Primer sequences used for gene expression analysis are shown in Table S1.

For GUS staining, 14 independent Pro*BnaC04.MYB89*::*GUS* transgenic lines were examined. A representative single-copy line was identified based on the 3:1 Mendelian ratio using glufosinate selection on the 1/2 MS medium with glufosinate-ammonium (10 mg/L). GUS staining was performed as reported by Jefferson [47], and the results were visualized under a bright-field microscopy (OLYMPUS SZX10, Tokyo, Japan).

### 2.8. ChIP-qPCR Assay

ChIP analysis was carried out with *B. napus* transgenic line *OE#11* following a protocol described in previous study [44]. Briefly, 4–5 g of the developing seeds at 30 DAP from K407 and *OE#11* plants was harvested for fixation. Chromatin was dissociated and sonicated to generate small DNA fragments ranging from 250 to 500 bp. The solubilized chromatins were immunoprecipitated overnight (≥10 h) at 4 °C using Anti-HA magnetic beads (Bimake #B26201), and co-immunoprecipitated DNA fragments were isolated and determined quantified using RT-qPCR with the SYBR qPCR Master Mix (Vazyme #Q711). The relative abundance of each candidate DNA fragment was calculated by normalizing the detected DNA amount to the *BnaGAPDH* genomic fragments (internal control) and subsequently to the corresponding input DNA samples, using *BnaACTIN7* as the negative control.

### 2.9. Morphological Observation of Seeds

The *BnaC04.MYB89* homozygous transgenic *A. thaliana* and *B. napus* lines, along with their corresponding wild-type plants, were cultivated under LD conditions. Mature seeds from the middle and base of the primary stems of different lines were collected, randomly selected, observed under a light microscope (OLYMPUS SZX10, Tokyo, Japan), and photographed.

### 2.10. Measurement of Fatty Acid in Seeds

To measure the fatty acids (FAs) in seeds, seeds from different *A. thaliana* and *B. napus* lines grown in separate pots were harvested. These seeds were taken from the middle and base of the primary stems of ten individual plants. The extraction and analysis of FAs were performed following the protocol from our prior study [48]. Briefly, 5–8 mg of the aforementioned seeds was subjected to extraction in a solution of methanol containing 2.5% (*v*/*v*) H_2_SO_4_ at 80 °C. Following a 2 h reaction, the FAs were converted into FA methyl esters (FAMEs). Upon reaching ambient temperature, the FAMEs were isolated with 1 mL of hexane and 2 mL of a NaCl solution (0.9% [*w*/*v*]), and the organic phases were collected and determined by gas chromatography (Shimadzu GC-2010 Plus, Kyoto, Japan), with methyl heptadecanoate (C17:0, NU-CHEK #CDDE-N-17-M) as the internal standard and the rapeseed oil reference mixture (AccuStandard #CDEZ-AOCS-007N) as the external standard. The peaks corresponding to each FA composition were identified by matching their retention times to those of the external standard, and the concentration of each FA compositions was then calculated relative to the internal standard. Finally, fatty acid composition data are expressed as micrograms (µg) of fatty acid per milligram (mg) of tissue dry weight.

### 2.11. Statistical Analysis

A completely randomized experimental design was carried out in this study. Data were presented as the mean ± SD and analyzed through One-way ANOVA using the SPSS software (v17.0). Significant differences were assessed using a 2-tailed paired Student’s *t*-test with statistical significance defined as *p* < 0.05.

## 3. Results

### 3.1. BnaC04.MYB89 Shares Conserved MYB-like DNA-Binding Domains and 3D Structures with Other MYB89 Genes

**Figure 1:**
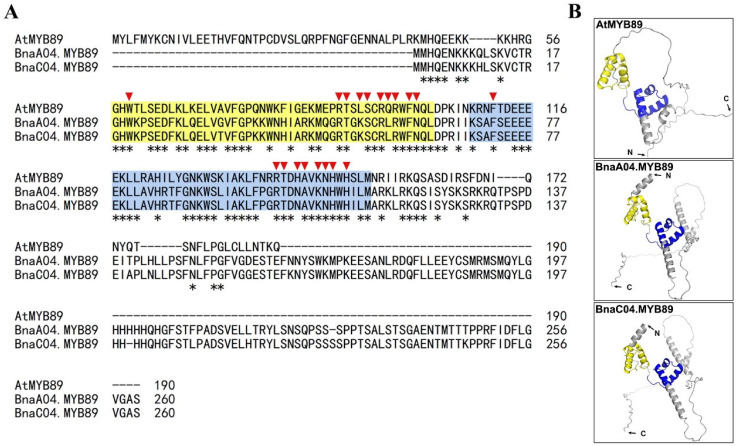
Analysis of the protein sequence and three-dimensional (3D) structure of three MYB89 proteins. (**A**) Protein sequence alignment of MYB89 from *Arabidopsis thaliana* and *Brassica napus*. Identical residues are indicated by asterisks. The two conserved MYB-like DNA-binding domains are highlighted in yellow and blue in the sequences, respectively. And the DNA-contact residues in the MYB-like DNA-binding domain are indicated by a downward red triangle. (**B**) Predicted 3D structure model of MYB89 proteins. The structural model was predicted with SWISS-MODEL and displayed with the Pymol software. The two conserved MYB-like DNA-binding domains are colored in yellow and blue, respectively.

We identified two BnaMYB89 genes in the ZS11 genome database (https://yanglab.hzau.edu.cn/BnIR accessed on 23 February 2023): BnaA04T0109500ZS and BnaC04T0392300ZS. They are located on chromosomes A04 and C04, respectively. These genes are designated as *BnaA04.MYB89* and *BnaC04.MYB89*. Sequences alignment revealed that the MYB89 proteins from *Brassica napus* and *Arabidopsis thaliana* shared two highly conserved MYB-like DNA-binding domains, along with the DNA-contact residues within these domains (Figure 1A). The *BnaC04.MYB89* gene is 783 bp in length and encodes a 260-residue protein, with an approximate molecular weight of 30.0 kDa. Amino acid sequence alignment showed that BnaC04.MYB89 shares 58.8% similarity with the AtMYB89 protein (Figure S1). Additionally, 3D structure analysis revealed that the three MYB89 proteins, particularly BnaA04.MYB89 and BnaC04.MYB89, exhibited similar structures characteristics (Figure 1B).

### 3.2. BnaC04.MYB89 Is Predominantly Expressed at the Seed Development Stage in Arabidopsis

**Figure 2:**
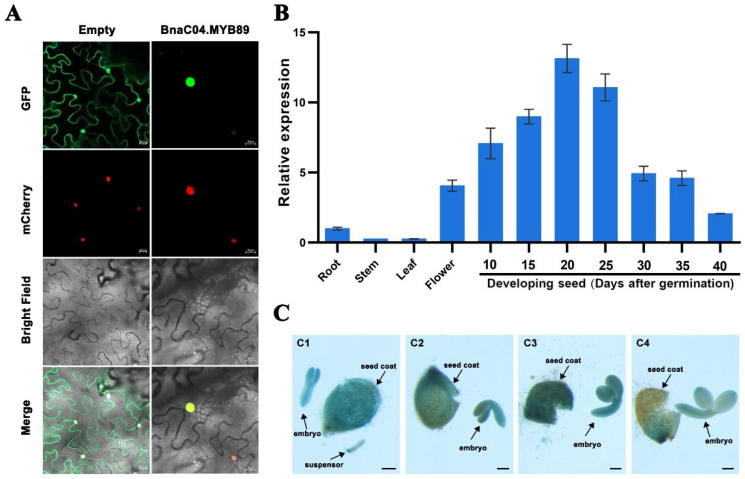
Analysis of *BnaC04.MYB89*’s expression pattern. (**A**) Subcellular localization of the BnaC04.MYB89 protein fused with GFP (35S::BnaC04.MYB89-GFP) in *N. benthamiana* leaves. mCherry, a nuclear localized protein fused with a red fluorescent protein; merge, merged with GFP, mCherry, and bright-field images. (**B**) RT-qPCR analysis of *BnaC04.MYB89* expression in various tissues (roots, stems, leaves, flowers, and developing seeds at 10, 15, 20, 25, 30, 35, and 40 DAP) of *B. napus* cultivar K407 plants. The RT-qPCR results were normalized against the expression of *BnaGAPDH* as an internal control. Values are means ± SD (n = 3). (**C**) GUS histochemical staining of Pro*BnaC04.MYB89::GUS* transgenic Arabidopsis plants shows the GUS expression level in developing seeds at 6 DAP (**C1**), 8 DAP (**C2**), 10 DAP (**C3**), and 12 DAP (**C4**). Scale bar = 100 μm. DAP, days after pollination.

To investigate the role of BnaC04.MYB89 in regulating seed fatty acid accumulation, we first examined its subcellular localization in tobacco leaves. The results showed that the BnaC04.MYB89-GFP fusion protein was predominantly observed in the nucleus, suggesting its functions as a transcription factor (Figure 2A). Next, we analyzed the stage- and tissue-specific expression patterns of *BnaC04.MYB89* in the *B. napus* cultivar K407 via RT-qPCR. The results indicated that *BnaC04.MYB89* was primarily expressed in both flowers and developing seeds (Figure 2B). During *B. napus* seed development, the expression of *BnaC04.MYB89* progressively increased during the early seed maturation stage, peaked at 20 DAP, and then declined. To further validate these findings, we generated Pro*BnaC04.MYB89*::GUS transgenic Arabidopsis lines in the Col-0 background and conducted histochemical analyses. As shown in Figure 2C, high GUS expression was observed in developing seeds, particularly in the embryos and seed coats during the early seed development stage.

### 3.3. BnaC04.MYB89 Represses Seed Fatty Acid Accumulation in A. thaliana

**Figure 3:**
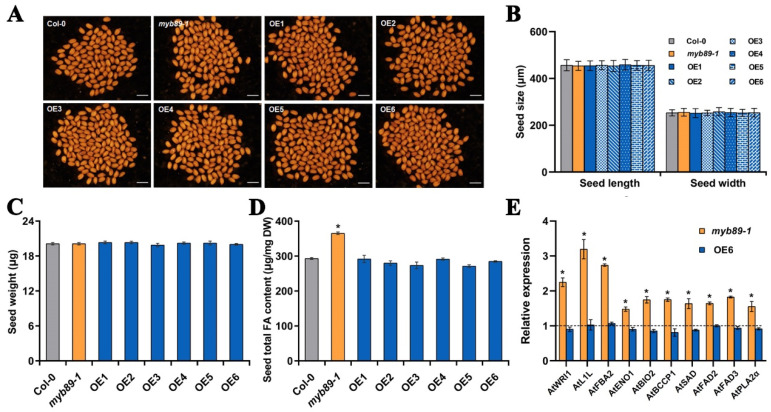
*BnaC04.MYB89* repressed seed fatty acid accumulation in *A. thaliana*. (**A**–**C**) Comparison of seed traits in various Arabidopsis lines. The seed traits, including seed coat color (**A**), size (Length and width, (**B**), and weight (**C**), were analyzed among Col-0, *myb89-1*, and the six transgenic Arabidopsis lines. Scale bar = 750 μm. (**D**) Total FA content in mature seeds of Col-0, *myb89-1*, and the six transgenic Arabidopsis lines. (**E**) Expression of genes contributing to seed fatty acid accumulation in developing seeds at 12 DAP in Col-0, *myb89-1*, and the transgenic Arabidopsis line *OE6*. Values represent means ± SD (n = 5), and each of the three assays for each biological replicate contained 300 seeds from 8 individual plants grown in different pots arranged randomly within one of three blocks. The RT-qPCR results were normalized against the expression levels of *AtEF1αA4* (the internal control). Asterisks indicate significant differences (*p* ≤ 0.05). DAP, days after pollination.

To preliminarily investigate the biological functions of BnaC04.MYB89 in seed fatty acid accumulation, we transformed the recombinant construct 35S::*BnaC04.MYB89*-6HA (based on the pGreen-6HA vector) into the Arabidopsis *myb89-1* mutant (Figure S2A). A total of eleven independent transgenic Arabidopsis lines were obtained following Basta selection. Among of them, six independent transgenic lines (*OE1*, *OE2*, *OE3*, *OE4*, *OE5*, and *OE6*) were further confirmed as homozygous through PCR amplification using the pGreen_F/BnaC04.MYB89_Rs primer pair (Figure S2B; Table S1). Meanwhile, the transcription level of *BnaC04.MYB89* was significantly higher in six transgenic Arabidopsis lines compared to Col-0 (Figure S2C). Subsequently, seed-related characteristics, such as coat color, size, and weight, were measured and compared. No significant differences were found in these traits among the mature seeds of Col-0, *myb89-1*, and six transgenic Arabidopsis lines (Figure 3A–C). Notably, overexpression of *BnaC04.MYB89* in the *myb89-1* background fully restored the high-FA-content phenotype observed in the *myb89-1* mutant (Figure 3D). To further explore whether BnaC04.MYB89 can regulate the FA biosynthetic genes that are markedly upregulated in *myb89-1* seeds [14], we performed RT-qPCR analysis using 12 DAP seeds from Col-0, *myb89-1*, and the transgenic Arabidopsis line *OE6*. The results showed that the transcription levels of ten FA biosynthetic genes, namely *AtWRI1*, *AtL1L*, *AtFBA2*, *AtENO1*, *AtBIO2*, *AtBCCP1*, *AtSAD*, *AtFAD2*, *AtFAD3*, and *AtPLA2α*, were significantly higher in *myb89-1* seeds, but they were restored to the Col-0 level in the seeds of the transgenic Arabidopsis line *OE6* (Figure 3E).

### 3.4. BnaC04.MYB89 Represses Seed Fatty Acid Accumulation in B. napus

**Figure 4:**
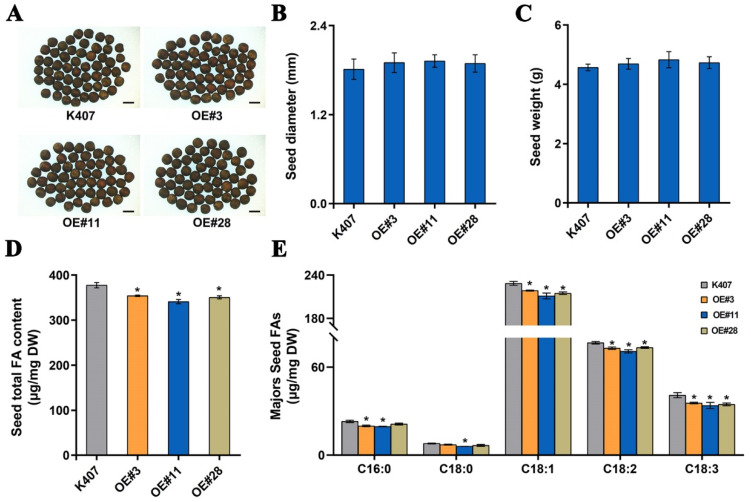
*BnaC04.MYB89* repressed seed fatty acid accumulation in *B. napus*. (**A**–**C**) Comparison of seed traits in various *B. napus* lines. The seed traits, including seed coat color (**A**), diameter (**B**), and weight (**C**), were analyzed from both wild type samples (K407) and the transgenic *B. napus* lines (*OE#3*, *OE#11*, and *OE#28*). Scale bar = 2 mm. (**D**,**E**) Total FA content (**D**) and the major FA compositions (**E**) in mature seeds in K407 and the transgenic *B. napus* lines (*OE#3*, *OE#11*, and *OE#28*). Values represent means ± SD (n = 5), and each of the three assays for each biological replicate contained 200 seeds from 8 individual plants grown in different pots arranged randomly within one of three blocks. Asterisks indicate significant differences (*p* ≤ 0.05). DAP, days after pollination.

To better elucidate the biological functions of BnaC04.MYB89 in seed fatty acid accumulation, we introduced the recombinant construct 35S::*BnaC04.MYB89*-6HA (based on the pCAMBIA1300-6HA vector) into the *B. napus* cultivar K407 (Figure S3A). Ten independent overexpression transgenic *B. napus* lines were obtained through hygromycin selection. Three independent homozygous transgenic lines (*OE#3*, *OE#11*, and *OE#28*) were confirmed through PCR amplification using the pCAMBIA_F/BnaC04. MYB89_Rs primer pair (Figure S3B; Table S1). The transcription level of *BnaC04.MYB89* was significantly higher in three transgenic lines than in K407, with *OE#11* exhibiting the highest expression among them (Figure S3C). Similar to the transgenic Arabidopsis lines, the transgenic *B. napus* lines displayed no alterations in seed coat color, diameter, or weight compared to K407 seeds (Figure 3A–C and Figure 4A–C). To assess the effect of BnaC04.MYB89 on seed FA accumulation, we analyzed the levels of five major FAs in the mature seeds of K407 and three transgenic *B. napus* lines. These FAs included palmitic (C16:0), stearic (C18:0), oleic (C18:1), linoleic (C18:2), and linolenic (C18:3) acids. The transgenic *B. napus* lines exhibited lower total FA content and an altered FA composition relative to K407 plants (Figure 4D,E).

### 3.5. Fatty Acid Biosynthesis and Metabolism Genes Are up- and DownRegulated in Transgenic B. napus Lines

**Figure 5:**
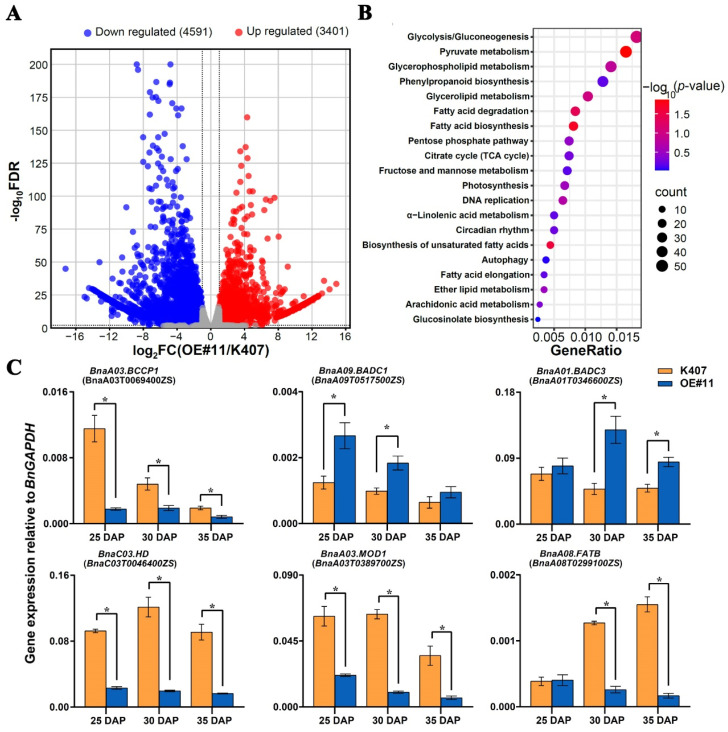
Transcriptome analysis. (**A**) Volcano plots of the differentially expressed genes (DEGs) in developing seeds of the transgenic *B. napus* line *OE#11* compared with those of the wild type (K407). (**B**) Gene Ontology (GO) enrichment analysis of DEGs. (**C**) RT-qPCR analysis of the expression of DEGs involved in seed fatty acid accumulation in the developing seeds of K407 and the transgenic *B. napus* line *OE#11* at three critical stages of seed fatty acid accumulation (25, 30, and 35 DAP). Values are means ± SD (n = 3). The results of RT-qPCR were normalized against the expression levels of *BnaGAPDH* (the internal control). Asterisks indicate significant differences (*p* ≤ 0.05). DAP, days after pollination.

Considering that the 30 DAP stage is critical for *B. napus* seed development, seeds from both K407 and the transgenic *B. napus* line *OE#11* at this developmental stage were selected for RNA-seq analysis to investigate the regulatory mechanism of BnaC04.MYB89 in seed FA biosynthesis and metabolism. A total of 47,994 genes were identified with 7992 differentially expressed genes (DEGs), including 3401 upregulated and 4591 downregulated genes (Figure 5A; Tables S2 and S3). GO analysis indicated that the DEGs were predominantly involved in FA biosynthesis and metabolic processes (Figure 5B). To validate the genes related to FA biosynthesis and metabolism identified in the RNA-seq analysis, RT-qPCR was performed to confirm their expression levels in developing seeds from K407 and *OE#11* at 25, 30, and 35 DAP. *BCCP1*, *HD*, *MOD1*, and *FATB* are positive regulators of FA biosynthesis [49,50,51,52]. Consistent with the previous studies, RNA-seq and RT-qPCR results also further verified that the expression levels of *BnaA03.BCCP1* (BnaA03T0069400ZS), *BnaC03.HD* (BnaC03T0046400ZS), *BnaA03.MOD1* (BnaA03T0389700ZS), and *BnaA08.FATB* (BnaA08T0299100ZS) were significantly lower in *OE#11* plants at all three developmental stages (25, 30, and 35 DAP) compared to K407, except for *BnaA08. FATB* at 25 DAP (Figure 5C). Additionally, we found that two *BIOTIN ATTACHMENT DOMAIN-CONTAINING* (*BADC*) genes, *BnaA09.BADC1* (BnaA09T0517500ZS) and *BnaA01.BADC3* (BnaA01T0346600ZS), which are known to inhibit ACC activity by competing with BCCP in Arabidopsis [53,54], were markedly upregulated in *OE#11* plants.

### 3.6. BnaC04.MYB89 Binds to BnaA03.BCCP1 and BnaC03.HD Promoters

**Figure 6:**
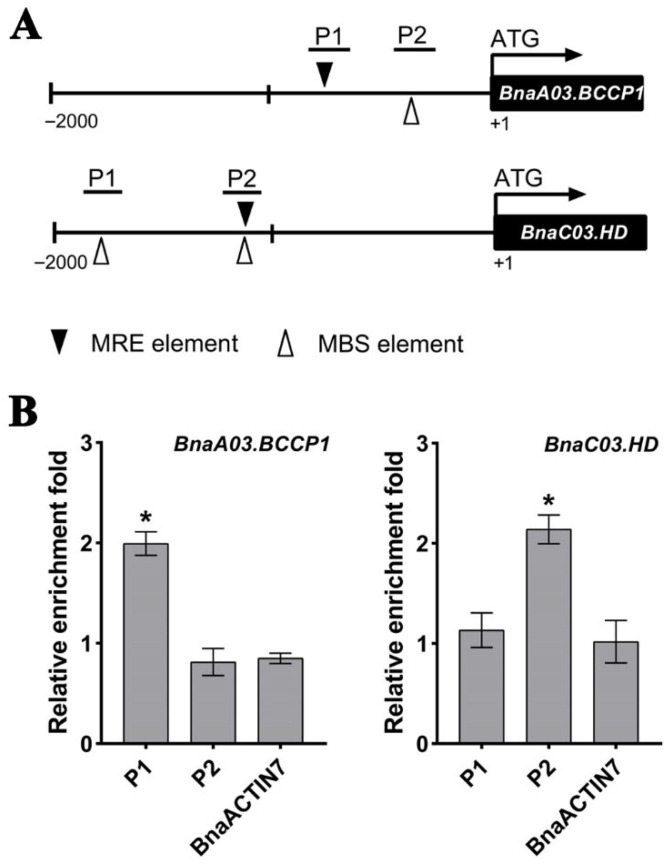
BnaC04.MYB89 bound to the promoters of *BnaA03.BCCP1* and *BnaC03.HD*. (**A**) Schematic diagrams illustrating the promoter regions of *BnaA03.BCCP1* and *BnaC03.HD*. (**B**) ChIP-qPCR analysis of BnaC04.MYB89-6HA binding to the promoter regions of *BnaA03.BCCP1* and *BnaC03.HD*. The fold enrichment of each fragment was calculated by normalizing the amount of a target DNA fragment against the genomic fragment of *BnaGAPDH* as an internal control, and then normalizing the value for the transgenic *B. napus* line *OE#11* against that of the wild type (K407). The *BnaACTIN7* fragment was used as the negative control. Values are means ± SD (n = 3). Asterisks indicate significant differences (*p* ≤ 0.05).

MYB proteins are known to specifically bind to MYB-binding core elements (MRE, AACCaaa; MBS, AA(A/C)AAAC) [55,56]. So, we examined the 2000 bp promoter regions of the aforementioned genes and found at least one MRE or MBS motif (Figure 6A and Figure S4A). Subsequently, we performed a ChIP-qPCR assay on those regions using seeds from the transgenic *B. napus* line *OE#11* at 30 DAP. ChIP-qPCR results revealed that BnaC04.MYB89 bound to the P1 region of *BnaA03.BCCP1* and the P2 region of *BnaC03.HD* (Figure 6B), while no binding was detected at the promoters of *BnaA03.MOD1*, *BnaA08.FATB*, *BnaA09.BADC1*, or *BnaA01.BADC3* (Figure S4B).

## 4. Discussion

Fatty acids (FAs) that accumulate as triacylglycerols (TAGs) in plants seeds are a major source of nutrients for humans and the main energy reserve to support seed germination and seedling growth [57,58,59]. Consequently, their accumulation is closely associated with the total crop yield. Given this critical role, enhancing FA accumulation in oilseed crops is a central breeding objective. As the world’s second-largest globally cultivated source of edible vegetable oil, *B. napus* represents a promising target for achieving substantial increases in seed oil content [6]. The seed oil of traditional *B. napus* cultivars is composed of 48–52% erucic acid (C22:1), 18–20% oleic acid (C18:1), 12% linoleic acid (C18:2), ~8% linolenic acid (C18:3), ~4% palmitic acid (C16:0), and ~2% stearic acid (C18:0) [60]. Consequently, this composition renders it unsuitable for food use. In contrast, modern “double low” (low erucic acid and glucosinolates) cultivars exhibit a substantially higher C18:1 content (~60%) and negligible erucic acid (C22:1) levels (below 1%) [3,4,60]. Given this optimized profile, the genetic materials derived from these “double low” cultivars provide a powerful foundation for elucidating the molecular mechanisms that control oil accumulation and fatty acid composition. To this end, we utilized the winter-type inbred line ‘K407’, a widely adopted double-low restorer line from the Shaanxi Hybrid Rape Research Center. This line was selected because it possesses two key attributes: well-documented high transformation efficiency for genetic studies [41,61,62] and an established role in commercial hybrid breeding [63,64]. Therefore, K407 serves as an ideal starting material for breeding high-quality rapeseed.

Seed oil accumulation is coordinately modulated by diverse factors, including external environmental and internal developmental signals, through an intricate network [8,65]. In this context, transcriptional regulation acts as a key node that orchestrates multi-level signaling networks, ultimately influencing oil accumulation processes [14,30]. However, the specific functions and underlying mechanisms of most transcription factors modulating seed oil accumulation remain largely elusive, highlighting the need for further in-depth investigation.

Oil in *B. napus* seeds starts to accumulate around 20 DAP in embryos, followed by a rapid accumulation period after 30 DAP [6]. Strikingly, the expression of *BnaC04.MYB89* preceded this process, increasing gradually from 10 DAP and peaking at 20 DAP before declining (Figure 2B,C). This early and transient expression pattern points to its potential role as a brake on the onset of accumulation. This hypothesis is supported by the observed dynamics of FA content in developing seeds, which only increased slowly at the early stage before accelerating rapidly 26 days after anthesis (DAA) and reaching its maximum at 38 DAA [27]. Furthermore, the expression pattern of *BnaC04.MYB89* is very similar to that of many key FA-biosynthetic genes, like *BCCPs*, *KASs*, and *HDs* [11]. Moreover, the expression pattern of *BnaC04.MYB89* is very similar to that of some vital FA modulators, including BnWRI1 and BnLEC1 [66]. Some modulators also share downstream regulatory targets with BnaC04.MYB89, like BnWIN1 and BnTT2 [24,67], further integrating it into the core regulatory network. Most conclusively, and consistent with its repressive function, the overexpression of *BnaC04.MYB89* led to a significant reduction in FA content and the expression of FA-related genes. We therefore posit that its knockdown or mutation would be expected to enhance FA accumulation. Collectively, our findings establish BnaC04.MYB89 as a negative regulator that fine-tunes FA biosynthesis during early seed development. In a future study, delineating the interactions among these modulators and BnaC04.MYB89 is necessary to fully understand the precise mechanism mediating fatty acid accumulation.

Research on MYB89 has primarily focused on seed development [14]. Recently, it has been demonstrated that ArMYB89 significantly promotes anthocyanin accumulation under low-temperature and low-light conditions in red maple [68]. Based on this finding, we hypothesize that a functional conservation may exist and that BnaC04.MYB89 could similarly modulate fatty acid accumulation in response to hormonal or environmental signals.

The de novo FA biosynthetic pathway has been extensively characterized in *A. thaliana* [11]. Four critical metabolic steps, namely condensation, reduction, dehydration, and a second reduction, act in a coordinated manner to determine the final seed oil content and FA composition in plants [9]. Transcription factors modulate seed oil accumulation by regulating the FA biosynthetic pathway [69]. Transcriptomic analysis of *B. napus* seeds indicates that orthologs of these FA biosynthetic genes and TFs maintain conserved functions that modulate oil accumulation and FA composition in *B. napus* [70]. However, few seed oil content- or FA-related genes in *B. napus* have been functionally studied. The overexpression of *BnaC04.MYB89* rescued the high-oil phenotype of the *myb89-1* mutant to the Col-0 level (Figure 3A–D). The high expression levels of regulatory genes (*AtWRI1* and *AtL1L*) and biosynthetic genes (*AtFBA2*, *AtENO1*, *AtBIO2*, *AtBCCP1*, *AtSAD*, *AtFAD2*, *AtFAD3*, and *AtPLA2α*) were also rescued to the Col-0 level (Figure 3E). These results suggest that BnaC04.MYB89 may exhibit comparable functional properties to AtMYB89 in modulating seed oil accumulation in *A. thaliana*. And this can be explained by the existence of the conserved domain between AtMYB89 and BnaC04.MYB89 (Figure 1). It makes more sense that the overexpression of *BnaC04.MYB89* can modulate oil accumulation in *B. napus* with an already high-oil genetic background because oil content in the major rapeseed cultivar seeds nowadays is generally much higher than that in *A. thaliana* seeds (Figure 4) [27]. Furthermore, BnaC04.MYB89 directly regulated *BCCP1* expression, like AtMYB89 (Figure 5), but other FA-related genes influenced by BnaC04.MYB89, *BACD1*, *BADC3*, *HD*, *MOD1*, and *FATB* were different from those of AtMYB89, which may be due to the different genetic background (Figure 5). In addition, the creation of high-oil germplasm through the knock-out of *BnC04.MYB89* can be carried out in a future study, which may provide a valuable reference for the cultivation of high-oil *B. napus* and other crops.

## 5. Conclusions

In summary, BnC04.MYB89 directly repressed the expression of *BnaA03.BCCP1* and *BnaC03.HD* and indirectly regulated the expression of *BnaA09.BADC1*, *BnaA01.BADC3*, *BnaA03.MOD1*, and *BnaA08.FATB* in developing seeds of the double-low restorer line K407, consequently modulating the fatty acid profile and ultimately inhibiting seed fatty acid accumulation. Our study verified that BnC04.MYB89 is a key regulator of both seed oil quantity and quality, thereby providing a genetic basis for cultivating high-oil *B. napus* and other crops through biotechnological approaches.

## Data Availability

The raw RNA-seq data generated in this study have been deposited in the NCBI Sequence Read Archive (SRA) under the BioProject accession number PRJNA1356366.

## Supplementary Materials

The following supporting information can be downloaded at https://www.mdpi.com/article/10.3390/plants14223495/s1, Figure S1: Pair-wise comparison of full-length amino acid sequences among AtMYB89, BnaA04.MYB89, and BnaC04.MYB89 proteins performed with DNASTAR Laser gene 11. Figure S2: Characterization of 35S::*BnaC04.MYB89*-6HA transgenic Arabidopsis plants. Figure S3: Characterization of 35S::*BnaC04.MYB89*-6HA transgenic *B. napus* plants. Figure S4: BnaC04.MYB89 could not bind to the promoters of *BnaA03.MOD1*, *BnaA08.FATB*, *BnaA09.BADC1*, and *BnaA01.BADC3*. Table S1: Primers used in this study. Table S2: A list of upregulated DEGs. Table S3: A list of downregulated DEGs.
