# Supplementary material for: Transcription Factor BnaC04.MYB89 Negatively Regulates Seed Fatty Acid Biosynthesis in Brassica napus
Source: Plants (Basel). 2025 Nov 16;14(22):3495. doi: 10.3390/plants14223495 (PMC12656447; doi:10.3390/plants14223495)
Supplement: Supplementary file 1 [file plants-14-03495-s001.zip › plants-3959546-supplementary/Supplementary data (Figure S1-S4).pdf]

Supplementary Figures

AtMYB89			
BnaA04.MYB89	58.8		
BnaC04.MYB89	58.8	96.5	
	AtMYB89	BnaA04.MYB89	BnaC04.MYB89

Fig. S1. Pair-wise comparison of full-length amino acid sequences among AtMYB89, BnaA04.MYB89, and BnaC04.MYB89 proteins performed with DNASTAR Lasergene 11. Sequence identity is given in %.

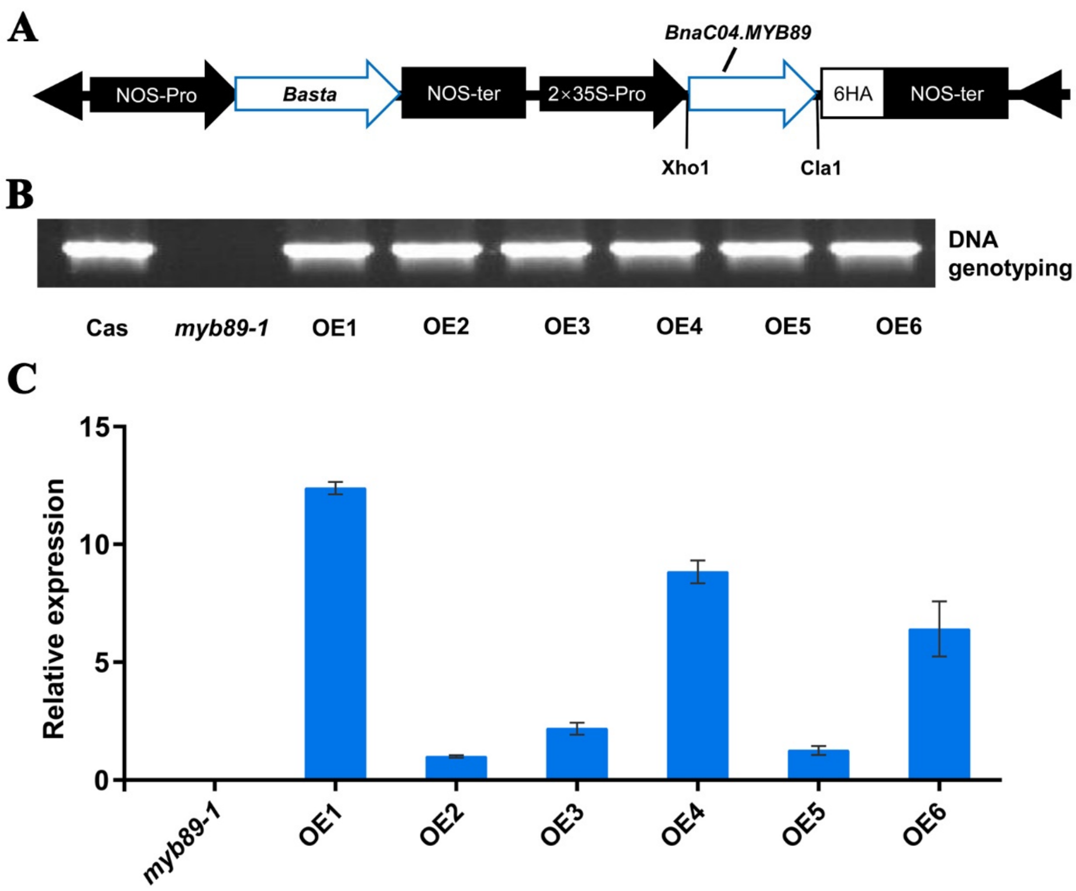

Fig. S2. Characterization of 35S::*BnaC04.MYB89*-6HA transgenic Arabidopsis plants. **(A)** Schematic diagram of the constitutive expression cassette of the *BnaC04.MYB89* gene in the binary vector pGreen-35S-6HA used for Arabidopsis transformation. RB, right border; LB, left border; NOS-pro, nopaline synthase promoter; NOS-ter, nopaline synthase terminator; Basta, glyphosate; 35S-pro, CaMV 35S promoter. **(B)** PCR-based DNA genotyping of wild type (Col-0), *myb89-1* and 35S::*BnaC04.MYB89*-6HA transgenic Arabidopsis plants using specific primers for the pGreen_F/BnaC04.MYB89_Rs. Cas, cassette. **(C)** RT-qPCR analysis of *BnaC04.MYB89* expression in one-week-old Col-0, *myb89-1* and 35S::*BnaC04.MYB89*-6HA transgenic Arabidopsis plants.

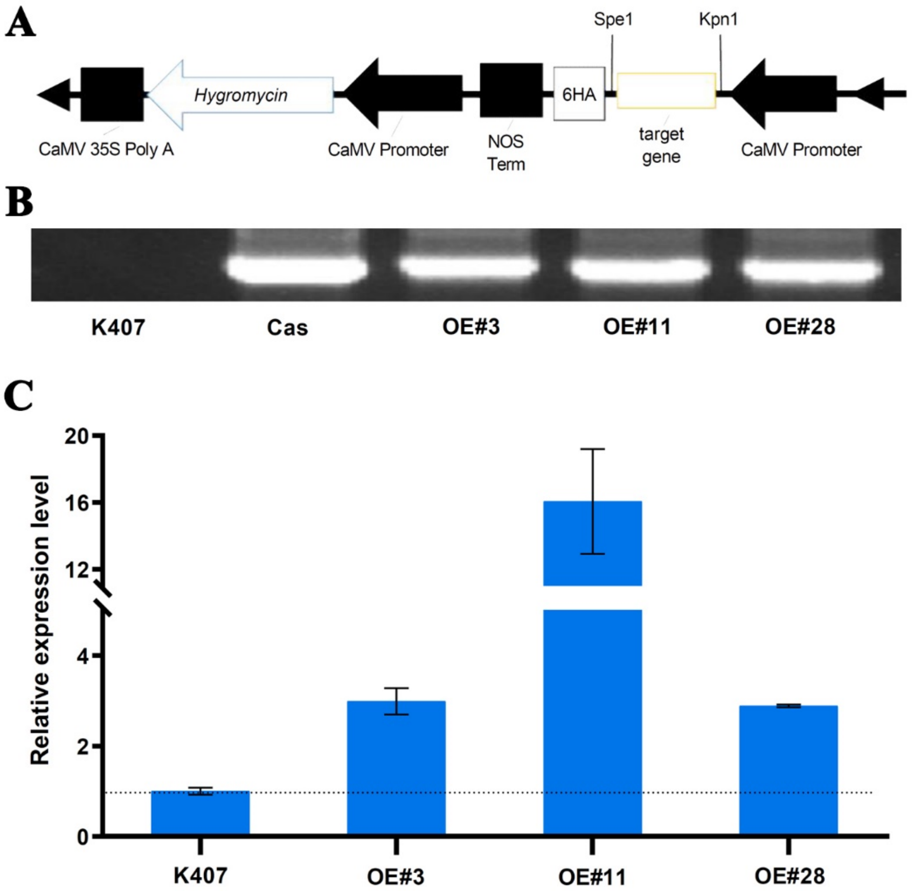

Fig. S3. Characterization of 35S::*BnaC04.MYB89*-6HA transgenic *B. napus* plants. **(A)** Schematic diagram of the constitutive expression cassette of the *BnaC04.MYB89* gene in the binary vector pCAMBIA-35S-6HA used for *B. napus* transformation. RB, right border; LB, left border; NOS term, nopaline synthase terminator; CaMV promoter, CaMV 35S promoter; Hygromycin, hygromycin resistance gene. **(B)** PCR-based DNA genotyping of the *B. napus* cultivar K407 and 35S::*BnaC04.MYB89*-6HA transgenic *B. napus* plants using specific primers for the pCAMBIA_F/BnaC04.MYB89_Rs. Cas, cassette. **(C)** RT-qPCR analysis of *BnaC04.MYB89* expression in K407 and 35S::*BnaC04.MYB89*-6HA transgenic *B. napus* plants.

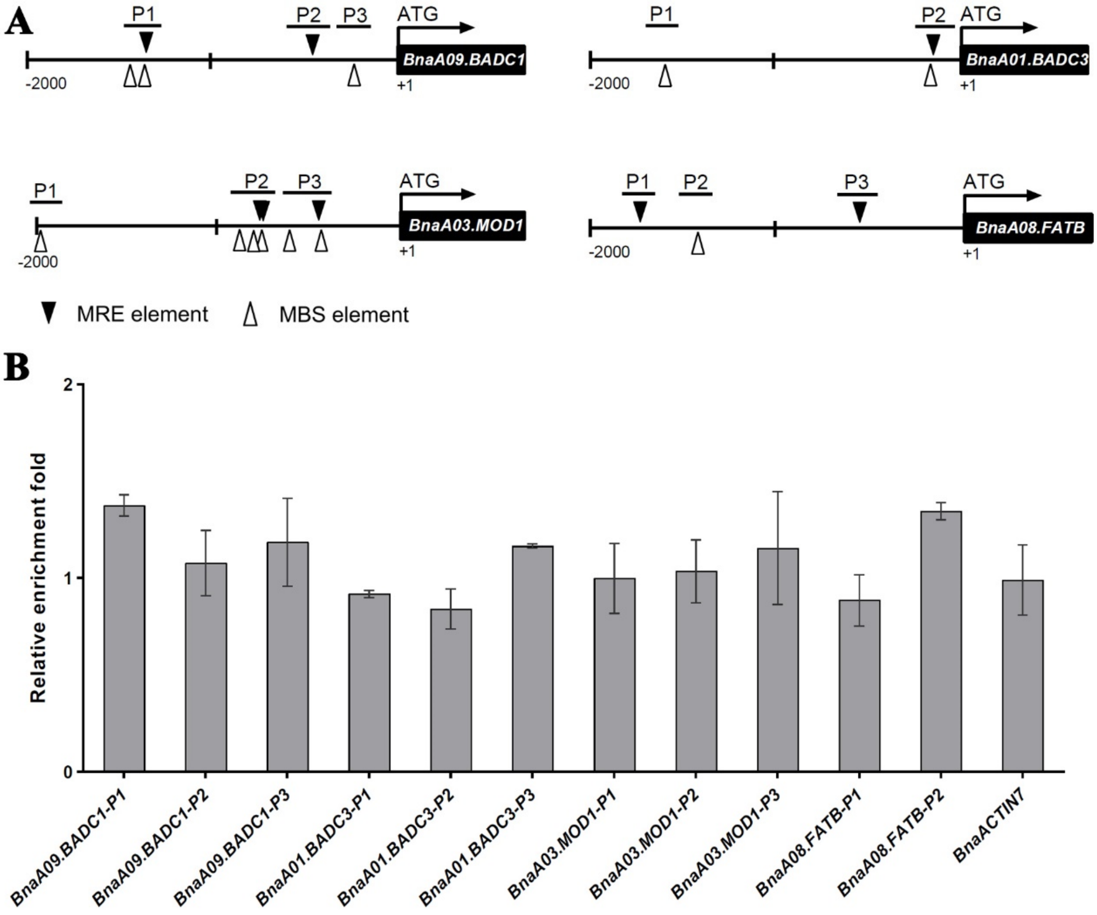

Fig. S4. BnaC04.MYB89 could not bind to the promoters of *BnaA03.MOD1*, *BnaA08.FATB*, *BnaA09.BADC1*, and *BnaA01.BADC3*. **(A)** Schematic diagrams illustrating the promoter regions of *BnaA03.MOD1*, *BnaA08.FATB*, *BnaA09.BADC1*, and *BnaA01.BADC3*. **(B)** ChIP-qPCR analysis of BnaC04.MYB89-6HA binding to the promoter region of *BnaA03.MOD1*, *BnaA08.FATB*, *BnaA09.BADC1*, and *BnaA01.BADC3*. The fold enrichment of each fragment was calculated by normalizing the amount of a target DNA fragment against the genomic fragment of *BnaGAPDH* as an internal control, and then normalizing the value for *OE#11* against that of the K407. The *BnaACTIN7* fragment was used as a negative control. Values are means ± SD (n = 3).
